# Questions in Medical Jurisprudence, Based on the Civil and Criminal Laws of Italy

**Published:** 1840-01

**Authors:** 


### Art. II.

*Questioni di Medicina Legale, secondo lo spirito delle Leggi Civili e Penali veglianti nei Governi d'Italia.* Del Dottore Giacomo Barzellotti, già P. P. di Medicina Legale, ec. nell Imper. e Reale Università di Siena. Ediz. 3a Pisana e 7ma Italica. T. iii.—*Pisa*, 1835-6. 3 vols. 8vo, pp. 493, 704, 514.

*Questions in Medical Jurisprudence, based on the Civil and Criminal Laws of Italy.* By Dr. G. Barzellotti, formerly Professor of Legal Medicine in the University of Siena, &c.—*Pisa*, 1835-6.

It is now twenty years since Professor Barzellotti first published a work on Medical Jurisprudence: that work, after having passed through many editions with various improvements, has served to form the basis of the present voluminous treatise. The author has followed the example of his great countryman, Zacchias, in attaching the modest title of "Questions" to this monument of his labour and industry; and, although we cannot dispute its appropriateness, yet we think few of our readers would form an idea from it of the extended and systematic manner in which all subjects purely medico-legal, and others relating to medical police, are treated. As it is a work which may not readily find its way into the hands of English practitioners, and one that relates to a very important, although, in this country, a still much neglected branch of medical education, we shall consider it our duty to furnish the readers of this Journal with a full review of the author's opinions on subjects that have any practical bearing. That the work is adapted to Italian jurisprudence will be no obstacle to this course; since, as the Professor judiciously remarks in his preface, "customs and laws may change, but facts are immutable:" so we say of medical jurisprudence, that the laws of nations may vary, but the well-established doctrines of the science will be the same in every civilized country. Thus the medical proofs of the crimes of infanticide and poisoning are the same in Italy as in England, although the laws of the two countries may define the crimes differently; and one may require a greater amount of evidence to establish legal guilt than the other.

No particular arrangement has been followed in treating the subjects, a circumstance which will not excite surprise, when it is considered that medico-legal questions may be viewed under very different aspects, according to circumstances; and that there is scarcely a case in which we do not find questions put to witnesses, that relate to subjects having no connexion whatever with each other. It is only then in the broad outlines of the science that a medical jurist can pretend to classify what falls before him; if he attempt more than this, he will, like many writers of the German school, sacrifice perspicuity to method. In the first volume, among many subordinate matters, the following subjects are examined: pregnancy, abortion, infanticide, and insanity; the second is entirely devoted to toxicology; and the third is equally divided between the subjects of wounds and medical police in relation to contagion and quarantine.

1. We pass over the author's remarks on age and sex, merely observing that they present all that is now known on those subjects. The questions relative to CRIMINAL ABORTION then offer themselves; and here we find a full exposition of the views of modern medical jurists. The causes of abortion and its effects on the mother and child are well described. In relation to the practice of inducing premature delivery, the author observes:

"Another question here presents itself; it is this: whether abortion may be lawfully brought on in medical practice, to prevent the risk which a female with a deformed pelvis may incur, by allowing her to go on to the full period? There is no question in legal medicine which, in a humane, moral, or legal point of view, is of greater importance than this, none which more closely concerns the honour of medical practitioners. But, however difficult this question may at first sight appear, from its being doubtful whether the mother should be saved to the exclusion of her child, the difficulty is not lessened when we consider that we have no means of determining at what period of gestation the child will have reached a stage of development, beyond which it cannot pass without endangering the life of the female. It matters not whether the pelvimeter has been applied to determine the diameters of the openings; because, as it is impossible to measure the head of the child, the application of this instrument can lead to no useful result, not to mention that there are other objections to its employment. Again, it can avail nothing to calculate from the period of pregnancy the degree of development in a child's head, and apply the inference to a particular case, since the heads of children are not of equal size at the same period: some are large, others are small, and, while in some the fontanelles are expanded, in others they are contracted. Moreover, even admitting that the narrowness of the outlets in a pregnant female, determined by the pelvimeter, were such that she could not give birth to a moderately-developed child without danger to the lives of both, I am totally opposed to the practice of inducing premature delivery, which professes to save the life of the mother and sacrifice that of the child. The reasons for my opposition to this practice are as follow: By its adoption, while there would be the almost certain loss of the life of the child, there is no certainty of saving the female, knowing, as we do well by experience, that abortion, maliciously induced by any means whatever, has always more or less compromised the life of a woman; hence, even if the practice were not deemed inhuman, it would be unjustifiable; because it cannot ensure the life of that one of the two beings for whose sake it would be employed. Moreover, if the deformity of the pelvis be known to the parents or professional men, why has the marriage been permitted to take place? Why should children be begotten when they can only be allowed to reach a certain stage of development, and are not likely to survive their birth above a few seconds? But, since the Cæsarean section or symphysotomy is more certainly adapted to save the lives of both when the child is mature, than the use of abortives when the child is immature, so it follows that while the adoption of these means is lawful, the use of abortives for the purpose of exciting premature delivery is unlawful and criminal." (Vol. i. p. 115.)

All who are engaged in obstetric practice must regard this as a momentous question; and we have therefore thought it right to state the author's opinions in his own words. There are two circumstances which, it appears to us, a medical man has to take into consideration before he engages in this practice, and these are embodied in the objections of the author. 1. Is it impossible that the woman can be delivered in a natural way at the full period? 2. Is it not very probable that she herself may fall a victim to the means used to procure premature delivery? In regard to the first point, the Professor truly observes, that no measurement can reach the child's head, that the size of the head is subject to great variation; and hence, although we may have evidence of the deformity of the pelvis, we are not in a condition to say that delivery in a natural way would be impossible; since the head may be smaller than the average, and the effects of nature may accommodate it to the contracted dimensions of the pelvis. This is no speculative view: we think it must be within the experience of most men who have been extensively engaged in obstetric practice, to have met with cases in which, in spite of great deformity, delivery has taken place at the full period, with safety to the mother if not to the child. We do not say that this has been without great risk to the mother; but it must be remembered, as a matter of responsibility, that it is better this risk should arise in the course of nature, than that it should be brought on by the means of art. In a remarkable case which occurred to Dr. Lilburn, reported lately in a contemporary journal,* we have an instance of what the efforts of nature will sometimes accomplish. A woman, labouring under great deformity of the pelvis, was twice delivered in safety and the child survived. In a very large number of cases, we think the question here proposed could not be answered from medical data, for we should have a difficulty in saying that it was *impossible* for the woman to be delivered at the full period. But, admitting that there are some cases in which the deformity is palpably great, and, at the same time, every allowance is made for smallness of the child's head and the efforts of nature, it may become a question whether premature expulsion should be resorted to. If it were simply a question whether the life of the mother should be saved to the exclusion of that of the child, we see not the least ground

* Med. Gazette. Vol. ix., p. 933.

for hesitation: but this is not exactly the position in which we are placed. Most experienced accoucheurs admit with the author, that abortion, however brought on, is attended with great risk to the female; and that, if weak and delicate, she may, in spite of the best care, easily sink under its effects. The saving of the life of the child cannot be generally looked to, and, indeed, it commonly happens that the child is still-born. Thus, then, the question may be said to be limited to the probable consequences of the process on the life of the woman. It is in order to save her, or to give her a greater chance of safety, that the operation is undertaken: the practice would never be justifiable, either legally or morally, for the mere purpose of saving the life of the child. Barzellotti rests his views upon the results of those cases in which abortives have been *criminally* administered to pregnant females: but the supporters of the practice would scarcely deem this fair; since it is well known that poisonous substances are commonly given in large doses by ignorant persons, and the death of the female, with or without the expulsion of a dead child, is the almost certain consequence. In this country, we believe, it is the custom of practitioners to excite premature delivery by the exhibition of regulated doses of secale, and by rupturing the membranes. Although this may lessen the danger to which the female is exposed, yet we think most practitioners would say with our author, that the operation was attended with considerable risk. It may at first sight appear to be justifiable on the same grounds as a severe surgical operation; but the comparison can scarcely be admitted; since, when a surgical operation is performed, it becomes justifiable by its being rendered absolutely necessary, and the danger to life being imminent. Can it be said that this is the case with a female, the deformity of whose pelvis, on which the whole question of necessity rests, cannot be accurately determined, who is only half advanced through pregnancy, and whose health at the time may not be in the least affected by her condition? But in cases of considerable deformity, in which alone it appears to us, if at all, the operation would be justifiable, at what period is the practitioner to perform it? If he perform it at the earliest stage at which pregnancy is discovered, he certainly exposes the life of the woman to great danger, because it is only by a most violent shock to the system that the undeveloped embryo can be separated: if, in the latter stage,—his attempts may be fruitless, since the head may have already passed beyond that degree of development which would have enabled it to traverse the outlet; and he is thus placing his patient in a worse condition than if the course of nature had been allowed to continue. If a fatal result follow, a not unlikely circumstance, the death of the female would be ascribed to his interference. Baudelocque and Capuron, two most eminent obstetric practitioners, have condemned this practice of exciting premature labour as an outrage against the laws of God and man. This, perhaps, is going to an extreme; the destruction of a child at birth by craniotomy is not recognized by divine or human laws; but every accoucheur knows that it must be occasionally resorted to, and that without it, not merely the child but the mother must perish. When the practice is undertaken on pure motives and with a view to save one life at least, we cannot ascribe immorality to the act, however we may condemn the conduct of the practitioner as indiscreet and imprudent. But what says the law of

England on this subject? The late statute, 1 Victoria, c. 85, s. 6, does not make any exception in favour of medical practitioners; it leaves the question entirely open. To render a person guilty under it, it is necessary that the means to procure "miscarriage" should have been *unlawfully* applied or used: but what constitutes an *unlawful* application or use is not defined. A medical man, if charged with the crime, upon the fact of his having made the attempt on a pregnant female, would be required to establish the purity of his motives, and to justify his conduct by a reference to general medical practice. In the first point he might succeed, but in the second, his success, if we are to be guided by the published opinions of great authorities, would be somewhat doubtful. His conduct might not be deemed illegal, but censured as indiscreet. On the other hand, if the female were to die under the process, he might find himself in a more serious position. The justification of his conduct must be more clearly made out than we think in most cases it could be, or, otherwise, a verdict of manslaughter might be returned against him. Thus, then, under all circumstances, the practice of exciting premature labour is not merely hazardous to the life of the female, but, whether she live or die from the attempt, it may seriously involve the reputation of a practitioner. Nor is it any answer to this view that it is often adopted with impunity to the woman and without the knowledge of the attempt becoming known; for the imprudence and illegality of an action are not affected by the occurrence of a few successful instances, or by the secrecy with which it is performed.

Our author dwells on the prevention of marriage by parents when the pelvis is known to be deformed, and on the nonprocreation of children among parties who are aware of this condition; but while pregnancy may occur in those who are unmarried, and a deformed female, who is exposed to the chances of pregnancy, does not speculate on the anatomical relations of her pelvis, we are called upon to meet the case of a woman so situated, and not to say how, when the difficulty has occurred, its occurrence might have been prevented. He suggests symphysotomy or the Cæsarean section as lawful substitutes for the practice which he holds to be unlawful and criminal, observing that the adoption of either of these at the full period is calculated to save the lives of both mother and child. Symphysotomy has, we believe, met with but little favour from English practitioners; they have preferred craniotomy; and it appears to us the last would be in most cases required, even where the first had been performed: hence little could be gained by its adoption. As to the Cæsarean section, it has been almost uniformly unsuccessful in England, chiefly because it was performed at too late a period. English practitioners look to this, rather as a means for saving the child than the mother. It is at all events a formidable operation to undertake upon a living healthy woman, and one which, in its results, is likely to be attended with as much responsibility on the part of the practitioner as the exciting of premature labour. The experience of Englishmen does not warrant us in adopting Barzellotti's view with regard to the Cæsarean section, namely, that it is more certainly adapted to save the lives of mother and child than the practice which he condemns. To us it appears the operation places the mother in equal if not in greater danger, while it rarely offers a greater chance of preserving the child.

It would seem that manual means, by which we are to understand surgical or obstetric operations, are not often criminally resorted to in Italy for the purpose of procuring abortion.

"Pharmaceutical means are sought for, and great art is shown by the guilty person in endeavouring to render a medical practitioner an unconscious accessary to the crime. Thus, pregnant females or their accomplices will invent some story to induce the practitioner to bleed, to apply leeches, to administer an emetic or drastic purgatives, at the same time, perhaps, concealing from him their real condition. Sometimes, when they are not successful in their application, they will resort to these means of their own accord, or will take savine or other substances popularly regarded as abortives. Notwithstanding these attempts, gestation will sometimes continue; but if it should happen that abortion followed, then the case may be treated as criminal; and an important medico-legal question may arise, as to whether the abortion was really caused by the means employed. This question will require some consideration, since, as we know that the simple means above mentioned are often used by pregnant women without any mischief following, how are we to judge in a particular case that the expulsion was really due to their employment? Much circumspection is here demanded of the witness, since, without it, he will either aggravate the case against the guilty person or lead to an acquittal. The judgment of the witness must be based on numerous circumstances, as on the time at which abortion occurred; on the condition of the female; her mode of life, her constitution, her moral conduct; and on the means actually employed by her, if known. An acquaintance with these circumstances becomes necessary to enable him to .say whether, in his opinion, the abortion was or was not brought about by criminal causes." (Vol. i. p. 117.)

These remarks are judicious, and convey an important lesson; for although a substance may have been administered with intent to procure abortion, and abortion have followed its use, it is not a necessary consequence that the expulsion should have been caused by it. This is a conclusion at which, for want of proper reflection, we might hastily arrive, through the proneness of the human mind to connect an effect with some immediately antecedent condition, which may be, however, only apparently the cause. This error of judgment will be avoided by attending to the advice of the author. It is singular that in our own statute law it does not seem to have been contemplated, that any woman would attempt the crime on herself, for the words obviously refer to the attempt made by others. This, indeed, is what we generally find. There are few women who, without the assistance of an accomplice, have sufficient knowledge to make the attempt.

In remarking on the difficulty of determining whether abortion has taken place at the early stages of gestation, more especially where the embryo or fœtus has not been found, the analysis of the sanguineous discharge will serve to throw some light on the question. Thus the lochia may be confounded with the menstrual secretion; but, in the author's view, they may be distinguished by the fact that the lochial discharge contains a large quantity of fibrine, while the menstrual secretion contains but little. We cannot say that we consider this would be admissible as a proof in an English court of law.

But, supposing that we have the best evidence of abortion having taken place in a particular female, how are we to determine whether it arose from a natural or a violent cause? The last condition must be positively established before legal guilt can be fixed upon a party: the question will not be allowed to remain as a mere matter of suspicion or inference from circumstances. The answer will be made up by a reference to certain facts which are within the province of a jury and others which are purely medical.

"The fœtus may be found buried or concealed; the female may have taken violent medicines, such as drastic purgatives or emetics, without the advice of a practitioner: she may have applied leeches to her feet, have abstained from food, or have employed mechanical means for the purpose of rupturing the membranes and discharging the waters and fœtus. On the contrary, suspicion would be removed, and the influence of natural causes rendered probable, if the woman were weak and of a languid constitution; if she had suffered from previous disease or violent emotions, arising from the loss of friends or fortune; if she had been subject to copious discharges of blood from the uterus or other organs, to diarrhœa or dysentery; if she had experienced a fall, or had taken violent exercise: any of these moral and physical circumstances are well known to favour the separation of the fœtus from the womb. The proof will be rendered still more satisfactory if she have not taken any precautions to conceal her pregnancy or delivery." (Vol. i. p. 120.)

When the dead fœtus is found and the female is unknown, the only medical grounds for her discovery and identification will rest upon the opinion formed as to the time which the fœtus may have survived its birth, and the period during which it has been exposed. The degree of its development and the advance of putrefaction will here form the data for a medical opinion. If we can say how long a period has elapsed since the expulsion of the child, circumstantial evidence may lead to the discovery of the mother; but to establish the case against her, something more than medical evidence will be required. There must be such a correspondence of time and circumstances as to leave no doubt in the minds of the jury, not only that she has been delivered of a child, but that she was delivered of this particular child. If many days have elapsed, the obtaining of medical proofs by an examination of her person will be hopeless. Abortion may be feigned for the purpose of extorting money, but a careful examination would soon enable a practitioner to detect the imposition.

"So it will happen that a mass of blood, a mole, or a group of hydatids may be mistaken for an aborted fœtus; but if the female be married and live in good society, there will be no difficulty offered to its production. Objections are always made when there has been illicit intercourse, more especially if the cause of abortion have been violent and criminal. In conclusion, I must caution medical witnesses that they ought not to allow the cause of justice and humanity to suffer, merely because the criminal or accomplice happens to be a member of their own profession." (Vol. i. p. 122.)

From the satisfactory manner in which this subject is treated, we are a little surprised that two cases, in which medical evidence may be material, have escaped the author's notice. Thus, a female may imagine that she is pregnant, and be so reputed by the world, when she is actually labouring under ovarian dropsy or some other abdominal or uterine disease. This impression will especially exist and continue for many months, when the female, if unmarried, has exposed herself to the chance of pregnancy. It is easy to conceive that abortion might be attempted in such a case as this; and the crime is complete under the statute, although the means employed may not have succeeded in producing expulsion. The accused party may be placed on trial, and, supposing that the medical witness is able to prove either that the female is not pregnant, or was not pregnant at the time the attempt was made—what would be the result? It is reasonable to presume that the charge would fall to the ground, for there would be, as the law terms it, no *corpus delicti.* It is singular that the statute makes no mention of the attempt being made on a *pregnant* woman; it merely says "to procure the miscarriage of *any woman:*" but two cases were tried in 1838, in which the judges respectively decided that pregnancy in the female at the time of the attempt must be proved, or the charge could not be sustained. One of these cases was that of a medical man, charged with having attempted abortion by the performance of an operation; he was acquitted, because the female denied her pregnancy, and there was no positive evidence of the fact.

But another and more difficult case presents itself, where, with almost every sign of pregnancy, the woman is not pregnant, as where the uterus contains a mole or a group of hydatids. Abortion may be attempted here, but the woman may recover from the effects of the means employed. If the contents of the uterus be expelled, their production would at once announce the nature of the case; but if they should not be expelled, then a most difficult question will occur; for a certain diagnosis could not perhaps be made, and it is not improbable that the medical witness would depose to the female having been pregnant at the time. But in the event of a mole or hydatids, or a mass of blood having been expelled instead of a fœtus, is the proof of this an answer to the charge? If the words of the statute were taken strictly in a popular sense, the term "miscarriage" would apply to all of these cases. It remains to be seen, however, whether our judges would or would not restrict its legal signification to the expulsion of a *human fœtus.* In a case of non-expulsion, where a mole or hydatids exist, from the great difficulty of diagnosis, it is most likely a criminal would be proceeded against and punished just as if the woman were really pregnant, unless, by the lapse of time before trial, the presumption of pregnancy had been refuted.

2. The question of LEGITIMACY, which is next examined, loses much of its interest, when we consider how little our courts of law are guided by the opinions of medical witnesses on the subject of gestation. It is now admitted that pregnancy may be protracted beyond the average period in some females, and that there are few in whom the period is exactly the same. So again in contested cases, courts of law have been known to declare some children illegitimate that had been born at the average period, and others legitimate which had been born considerably beyond it. This is as it should be. Whatever medical opinions may be offered, and these we know are sufficiently conflicting, owing to each witness being commonly ruled by his own experience and rejecting that of his fellow-witnesses, the court always places the greatest reliance on moral proofs. It is, indeed, difficult to conceive a case in which if a witness were justified, from medical data, in pronouncing for illegitimacy, as where a child is said to have been born at the fourteenth or fifteenth month after intercourse between its reputed parents, there would not be, at the same time, an abundance of moral proofs to establish the fact conclusively, without the assistance of a single medical opinion.

3. In a country where the law of primogeniture prevails, the determination of the order of births, in a case where a woman is delivered of twins or triplets, becomes a matter of importance in relation to the civil rights of children. By the Roman law this is not necessary, since all children, born of the same father, have equal rights of succession; when, however, property is specially bequeathed to the eldest child, the same necessity exists for determining these cases, as where the law of primogeniture is in force. (p. 138.) This is a question which can rarely present itself, and we think when it does occur, the court would obtain the necessary evidence rather from parents and nurses than from medical witnesses. Prof. Barzellotti admits the doctrine of superfœtation, although we find no new cases in support of it; those which he adduces are well known to the profession, being quoted from the works of Orfila, Baudelocque, and Foderé. It appears to us that the author, in treating of this question, has confounded two conditions which ought to be held distinct; namely, of one conception following another either immediately or at an interval of some months. Those who are disposed to admit the first, that two conceptions may take place at or about the same time, may not feel disposed to believe the second, considering the well-known changes which take place in the uterus when the fœtus has reached the second, third, or fourth month of its development. Many instances have been reported in support of the last view, but to us the evidence does not appear satisfactory. In admitting the facts stated by the author and those whose opinions he adopts, we differ from him, in thinking that they allow of another explanation more probable and more consistent with the laws of the pregnant state than that assigned by the advocates of superfœtation. The only possible legal case where a question of this kind could be entertained in this country, is where the children, alleged to be of the same father, are born at different periods, we will say at three months' interval, and are equally mature and developed, as for instance, where a widow gives birth to two children after the death of her husband. These have in general been regarded, and very properly, as twin cases, in which one child was born before the other. The statement of the children having been equally mature at birth is not admissible, because it has rested on mere hearsay or ocular inspection, instead of being founded on the weight, length, and other characters of development. The author, however, maintains that one of the proofs of superfœtation is, that children may be born at the same time, presenting very different degrees of development, one will be large and robust, the other small and weak (p. 141); therefore, he argues, one must have been conceived after the other had become developed. We certainly are not in a condition to say at what particular period after the first impregnation, the power of conception ceases in a woman; but we should consider it a very unlikely occurrence after the first month; and we think that cases which are set down as superfœtations within this period are equally well explained by supposing that the children were really twins and one was developed more than the other. A bilocular uterus was supposed to be favorable to this condition, and the author advances the opinion, but there are some difficulties to its admission. A woman died after delivery, and on inspection, a bilocular uterus was found; but impregnation had only taken place in one cornu:* in another instance, a woman, in whom superfœtation was believed to have existed, died, and on dissection, the uterus was found in its normal state.† The practical application of this doctrine in jurisprudence is limited to those cases only in which moral and medical proofs would easily solve the question.

* Med. Gazette, Vol. xix., p. 507.

† Devergie, Vol. i., p. 469.

Such a case never has occurred, and does not appear likely to occur. The delivery of a woman of two *equally mature* children at intervals of two or three months is contrary to general experience. An artful woman might make use of the doctrine to substitute one child for another, as a male for a female, or a living for a dead child; but this is supposing her to be profoundly versed in the difficulties of legal medicine. The occurrence of such a case must be generally considered too improbable for women in ordinary life ever to have recourse to a sort of imposition which would be so easy of detection.

4. The chapter relative to the RIGHT OF SUCCESSION in new-born children is full of important matter; and we are pleased to find that the author takes a much more liberal and enlarged view of this subject than the medical jurists of France and Germany. He does not examine the question of "what constitutes birth," but in England this may give rise to grave enquiry. The practice of our law is not to consider a child as born, in civil or criminal cases, until the *whole* of its body is in the world. The establishment of respiration before birth amounts to nothing; the child must be entirely born and live *after birth* to entitle it to the rights of inheritance. On the other hand, to render proof of life after birth admissible in law, it is not necessary that the child should have breathed, its legal survivorship may be established by the evidence of other vital acts. On the continent, in general, before a child can take property by descent or bequest, it must not only be proved to have been born alive, but it must be proved to possess *viability*. Foreign jurists attach different meanings to this word; but the general interpretation seems to be that the child should have all its organs and functions perfect, and that there be no vice of conformation about it likely to lead to a speedy termination of life. According to some it must also be born at a certain period of gestation; its body must be of a certain length and weight, and present certain marks of development, or else its rights of inheritance will be disputed, and probably set aside in law. We may well ask what relation have all these accidents with the natural claim which every child has to inherit the property of its parents? We can only reply that they give rise to abundance of litigation and to the display of much legal and medical ingenuity, but the common-sense view of the subject is lost sight of in the purest fictions and technicalities. The Professor justly remarks, that the proofs of the act of life after birth, and of viability or the capacity to live, should be considered as perfectly distinct. (p. 178.) He very properly regards the division of life into uterine and extrauterine, or vegetative and animal, as absurd, if it be taken from what it properly belongs to, namely, speculative physiology, and applied to the practice of jurisprudence. There is no doubt of the fœtus possessing life while in the womb, and if at its entrance into the world any of its uterine functions be still carried on, this is satisfactory evidence of its being alive. Thus, "the persistence of the circulation after birth, as evidenced by the motions of the heart and arteries, ought to leave the question of the child being living undisputed; and we might even say that it possessed a greater share of vitality than it did in the uterus, because it can now perform certain actions which it could not while within that organ." (p. 179.) "Even where all pulsation has ceased and there is no sign of life, its death may be only apparent, for it may still under proper care be resuscitated." (p. 180.) The question of life being determined, the author passes to the examination of viability. Into this we shall not enter, since it is an exposition of the French doctrines on which we have already commented. Barzellotti contends that the establishment of respiration should not be deemed a necessary proof of viability, for, he observes, the lungs may be well formed, and the non-establishment of the process be due to some simple accident. The proof of circulation should be sufficient, without its usual concomitant respiration; and he very properly asks, "why should well-organized and living children be declared not *viable*, and deprived of their civil rights, because they happen to be in a state of apoplexy or asphyxia, when others (adults), affected with apoplexy or asphyxia, have not their rights denied them." (p. 183.) That courts should come to contradictory decisions, while such absurd points are insisted on as matters of proof, cannot excite surprise. Two examples are quoted from Zacchias, illustrative of this uncertainty of the law.

"A woman died during labour, and the child was extracted by the Cæsarean section. It moved its arms, legs, and mouth, so that those present had no doubt of its being alive. A division of opinion, however, arose, on the matter becoming litigated, whether this child was *viable*, i. e. endowed with capacity to live. Zacchias strongly maintained that it was not, because it had not come into the world in a natural way, but had been removed from its mother's womb by an operation. The Roman court decided in accordance with this opinion; but it is easy to perceive that their decision was based upon defects connected with the mother, not with the child, and that more weight was attached to the manner in which it came into the world than to the signs of life which it had really manifested." (p. 185.)

In another case, however, somewhat similar in its details, Zacchias gave an opinion in favour of the viability of a child, because it had reached maturity and had manifested *signs of life* after birth. The court again decided according to this view, and, therefore, in opposition to its former judgment.

The author next refers to the remarkable case of Fish v. Palmer, which was tried in our Court of Exchequer in 1806, not in 1796, as stated by him. Here the question was, whether the motion of the lips of a child after birth should be deemed evidence of *life*. Drs. Babington and Haighton gave an opinion in the affirmative, Dr. Denman in the negative. The jury decided that the evidence was sufficient to prove life, upon the same principle that dead muscle cannot spontaneously contract. Foderé condemned this decision, but our author upholds it with irrefragable arguments, although we think it requires but little reflection to perceive that the verdict was founded on the strictest equity. He is, however, in error when he says (p. 187) that the verdict declared the child to be *viable*, for the law of England knows nothing about the *viability* of children. Their capacity of continuing to live after birth never becomes a question. All that is required to be proved is that a child was not dead when born; and, as the above decision shows us, the least action, indicative of vitality, as the quivering of a lip, is sufficient to establish this point. Respiration, the act of crying, or the free motion of the limbs, will, *à fortiori*, be good evidence; but these actions are not indispensably required to be proved in order that a new-born child should take its civil rights. We gather from our author that the Italian law demands proof of viability besides evidence of life, although, at the same time, it construes the facts most leniently towards the child.

Thus it will not insist upon proof of all the functions of life being exercised by the offspring (p. 186); but we are entitled to ask, why should any proof beyond that of the possession of life after birth be required? The slightest reflection will show that the greatest possible injustice must be inflicted where proofs of viability are rigorously insisted upon; the opinions of medical witnesses cannot be founded on any fixed rules, and the decisions of courts of law under such legislation will always be arbitrary and often conflicting.

5. The chapter is closed by some remarks on the civil rights of MONSTERS. Continental codes of law are chiefly based, not so much on the degree of deformity in these beings as on their viability or capacity to live. Our English law is regulated by external form only: "a being to inherit must have *human* shape." Slight external defects, as a superabundance or deficiency of fingers or toes, do not debar from civil rights either in England or on the continent, still there is this remarkable difference between our laws and those of France and Italy:—according to the codes of the latter countries, monstrosity may be *internal* and cut off civil rights, as where there is a transposition or deficiency of important viscera, leading necessarily, and sooner or later, to the death of the being: but in the law of England, so far as we can judge from the highest authorities on the subject, the want or misplacement of internal organs cannot take away the civil rights of a child provided it be born with life. The author does not appear to have met with the excellent remarks of Geoffroy St. Hilaire on Teratology in relation to Medical Jurisprudence. He thinks, however, that both disomatous and dicephalous monsters should be allowed the rights enjoyed by other beings, and he adduces the case of Christina Ritta to show that they ought to be deemed viable, this compound monster having lived about eight months. He does not mention the Siamese twins, probably the most remarkable disomatous monster ever known, and one well calculated to baffle all legislation on the rights and responsibility of such beings. A case like that of the Siamese twins might give rise to a question more serious than one relating to the right of succession, as when, for instance, murder is committed by one half of the monster, without the criminal participation of the other. St. Hilaire quotes a case of this kind, which actually occurred in Paris, in the seventeenth century. A disomatous monster killed a man by stabbing him with a knife. The being was condemned to death, but was not executed, on account of the innocence of one of its component halves.*

6. We now pass to the questions connected with INFANTICIDE. The practitioner is first directed to determine the age and probable period of death of a child found dead, and particularly to attend to the phenomena of uterine putrefaction. The moment we have discovered that uterine putrefaction has taken place in the body of a new-born child, we do away with the charge of infanticide. The signs of putrefaction in utero are chiefly extracted from Orfila. In the Fourth Number of this Journal our readers will find a notice of the valuable remarks of Devergie on this subject. These comprise all that is known respecting it, and having so recently given a full exposition of them, it is unnecessary for us to quote those contained in the work before us. Respiration, in the

* Annales d'Hygiène. 1837. Vol. i. p. 431.

opinion of the author, is the chief character of extra-uterine life, an opinion in which we fully coincide, with the understanding that the function may have been performed and the child not have been born living; as also, that a child may have lived after birth, in some instances many hours, without leaving any traces of the performance of that process in the lungs. The test of Ploucquet, founded on the relative weight of the lungs to the body before and after respiration, is first examined; but the author places no reliance on it, chiefly from the conflicting results obtained by Schmitt and Chaussier. Although we think this test is not of much service in a practical view to the medical jurist, yet we are not led to this opinion by the statements of the writers on whom the author relies. When we know that to obtain these results they experimented on the lungs of children born at various periods of gestation and of both sexes, we can easily account for the wide difference between their ratios and those of Ploucquet. Experience clearly shows that the ratio is different according to the degree of maturity of the child and the period of gestation at which it is born.

The author seems inclined to place great reliance upon the complicated process, suggested by Professor Bernt, of Vienna, for solving the question of respiration in a new-born child found dead. The principles of Bernt's process are minutely described. This medical jurist takes the length of the body, the weight of the lungs and heart together, the weight of the lungs alone, as well as the specific gravity of these organs. Averages are first obtained by experimenting in this way on children of the two sexes and at different ages, which have respired perfectly, imperfectly, or have died without respiring, These results, in an unknown case, may, it is alleged, be employed as standards of comparison. The only objection to this process is, that if it were possible of application in the hands of practitioners, it would be inadmissible as evidence in a court of law. No accused party would be convicted upon mere averages, for how are we to be certain that we have obtained correct averages? The rules of law imperatively require that certainty and not probability should be adduced to establish a particular fact. When this certainty cannot be obtained, the court will prefer leaving the point undecided rather than incur the risk of deciding incorrectly. As a medical question, we would ask what relation can be suspected to exist between the *length* of the body of a child and the absolute *weight* of its lungs? Experience, so far as we are aware, does not show that there is any connexion between these conditions, and it would be very singular if there were: yet the author seems to admit, although not from any observations of his own, that there is a close relation between them. (p. 259.) Orfila showed that Bernt's conclusions were not supported by his own (Bernt's) experiments: but Barzellotti objects to Orfila's criticism, that he did not include the length of the bodies in the cases to which he refers. For reasons already stated, we are inclined to adopt Orfila's view of the insufficiency and inadequacy of the process suggested by the learned Viennese Professor.

The hydrostatic test is somewhat briefly disposed of. We find here introduced the novelty of connecting the weight of the lungs with the length of the child's body and comparing it with a standard, prior to drawing any inference from the sinking or floating of the organs in water. Thus, he observes, "if the lungs float without having an absolute weight equal to that of the lungs of children of *the same length*, which have respired, or which, by immersion, have not displaced so much water as these, there is reason to believe, either that they belonged to children which have died in utero, or that the air they contained was due to artificial inflation, putrefaction, or emphysema." (p. 273.)

The author has here departed from the usual practice. Most medical jurists look to the age or degree of maturity of the child before they draw an inference from the application of the hydrostatic test; but the degree of maturity does not depend upon the length of the child's body alone. This is only one of many characters, and consequently error is more likely to arise in this case than when all are taken. The following is a singular and to us a novel application of the test:

"Supposing the lungs to be absolutely heavier than the length of the body would indicate, and, at the same time, specifically heavier than water, while they are free from disease, so much air should be gently blown into them, by an opening made in the trachea, as the organs will conveniently admit. In the case here imagined, the lungs would weigh more than an ounce (the average before respiration), and would fall to the bottom of water. After this operation, the absolute weight need not be again taken, since it will not be altered by the introduction of air. Let the lungs thus inflated be placed in water, and let it be observed, whether the inflation has rendered them buoyant. Now, removing them from the vessel, so much blood, drawn from a living animal, should be injected into them, through the pulmonary artery, as they will receive; in the absence of blood, milk may be used. The pulmonary artery being again secured by a ligature, the lungs are to be accurately weighed and the difference between their present and former weight noted. They must then be put into water, and the quantity of water displaced by them measured. Let the results, thus obtained, be compared with those derived from the lungs of children that have been born, after having breathed, ("di feti nati dopo di aver respirato;") and if, after the inflation and injection, the condition of the lungs be the same in the two cases,—we are justified in asserting that the organs experimented on, had not respired (after birth); for by this process we place them under the same circumstances as those with which they are compared. A fraud may be in this way detected: thus, if lungs, which originally contained air, have been so compressed as that their buoyancy is destroyed, it may be discovered by inflation and injection; for the organs thus treated will then admit more air and more blood than those which have not really respired. The experiment is also applicable to cases in which the lungs are diseased." (Vol. i. p. 273.)

The Professor seems to be well aware of the fallacies to which this plan of determining the question of respiration is exposed. In admitting its ingenuity, we doubt how far it can be safely applied in practice, or even, if it were safely applicable, how far it would add to the utility of the processes already employed. The principle on which it rests is that the fœtal lungs cannot become so readily penetrated by air, and liquids resembling blood as those which have actually respired. But by what standard is the examiner to regulate the force of inflation and injection? If the fœtal lungs of two children were given to two experimentalists, it is quite possible, according to the force employed, that in one case the lungs would be brought to resemble those which had respired, and in the other, those which had not. We know, from actual experiment, that the fœtal lungs more readily receive air by inflation in some cases than in others, and this may easily occur in the injection of liquids, for the lungs of the fœtus contain variable quantities of blood, and, therefore, will admit variable proportions of injected liquid, without reference to the influence of the varying degree of force employed in the injection. If occurrences of this kind are only *possible,* how is such a test to be made practically available in legal medicine? Besides, we do not see that even were it otherwise, any advantage would be gained by resorting to it. The absolute weight of the lungs, coupled with their healthy appearance and the fact of their sinking in water, furnishes just as much evidence as could be obtained by adopting the processes here suggested. In so far as they appear to us exposed to error, they lead to one of a dangerous nature, namely, that of causing us to say that lungs may have breathed which have never received air by respiration.

In endeavouring to solve this question, we are further advised to notice if the thorax be flattened or not; also the position of the diaphragm, and the state of the abdominal viscera. These facts will enable us to decide whether the child had died during birth. "If a child has breathed, but dies before it is born, owing to narrowness of the outlets, this ought to be indicated by marks of violence on the body: if these should be wanting, there will be no other evidence of the fact." (p. 276.) We think that scarcely sufficient stress has been laid, in the examination of this question, on the difference between the evidence of life and of live birth in the child. All the proofs adduced go simply to show that a child has *breathed,* but not that it has been *born alive;* and a medical jurist ought always to treat these as matters essentially distinct. We are not informed whether the Italian law includes under the head of infanticide those cases in which a living child is destroyed during the act of birth. By the English law, this is not treated as child murder; our judges invariably holding, when the question is raised, that to constitute the crime, the whole of the body of the child must be in the world when the murderous violence is offered. We have already recorded our opinion that the English law is, in this respect, impolitic and bad.

We shall not follow our author in his account of the causes of death of the child, nor into the question of presumed survivorship, where mother and child are found dead. These subjects are treated with the care which their importance demands.

7. We next arrive at the description of the various forms of death by ASPHYXIA, as drowning, hanging, &c. Syncope and asphyxia are considered to be synonymous terms; and either is employed by the author to signify apparent death, though he thinks the word syncope the more applicable. The sign upon which he relies most as evidence of drowning is the presence of a bloody mucous froth in the trachea. A remarkable case is related in this section, which shows that much caution is required in drawing an inference from the presence of marks of violence on the drowned; we here furnish a brief abstract of it.

"A prisoner, while being escorted by a party of soldiers along the banks of the Po, took an opportunity of throwing himself into the river, for the purpose of escaping. His arms were at the time bound together; it was alleged that he had perished by drowning. Thirty-three days after the accident, the body was discovered, and submitted to a close examination. Besides the ordinary signs of drowning in the respiratory organs, some appearances were seen on the neck, which led to the suspicion of strangulation. There was a livid circle extending completely around the neck, about a line and a half wide; and immediately below it another mark, lighter in colour. The skin over the trachea was found to be ecchymosed. Blood was extravasated in the brain, and between its membranes. The presence of the marks, which some supposed to have originated from strangulation, was explained by the fact of the deceased having worn a thick coarse shirt, tightly buttoned round the neck. The water, it appears, caused by imbibition a retraction of the stuff, and thus made the shirt-collar act as a tight ligature." (p. 329.)

Barzellotti and Orfila gave it as their opinion that this was a case of drowning, and the persons who were charged with having strangled the deceased were set at liberty.

We knew of an instance where the body of a female who had perished by accidental drowning, off the coast of Norfolk, presented, as in the above case, a deep livid mark around the neck. This was found, by careful examination, to have been produced by the string of a cloak which the deceased wore at the time of the accident. She was not more than ten minutes in the water, and it was probable the mark had arisen from her struggling in one direction, while the tide was drifting her cloak in another. Supposing that the accident had not been witnessed; that the cloak had separated, and the body drifted on shore, what might have been the medical conclusion? It would perhaps have been said, how could a mark like this have resulted from accident?

We fully agree with the author that the question whether the drowning is suicidal or homicidal, is rather for the jury than the medical witness. It is not wonderful that a question of this kind should so seldom be raised in an English court of law, when we consider that coroners, to save time, trouble, and expense, generally direct the verdicts of juries from their own casual inspection of the body. They do not require medical evidence even to establish the real cause of death. A dead body having been found in the water is in general held to be a sufficient proof of death by drowning!

8. In treating of *hanging*, the author seems to be fully aware of the fallacies which may arise from trusting to the presence of a mark of the ligature on the neck, as evidence of vital hanging. Ecchymosis, even below the cord, cannot be trusted to as a proof of this, unless the effusion of blood extend beneath the skin and adjacent muscles. The subject of *suffocation* is shortly discussed. We were not aware before that the Emperor Tiberius fell a victim to the process of "burking," or that that form of murder was so ancient. (p. 335.) On the whole, the remarks on the subject of death from asphyxia, in its various forms, are practical and judicious.

9. The next chapter is entirely devoted to an exposition of the rules for conducting medico-legal inspections, and for drawing-up the necessary reports. The following observations plainly show that the customs of Italy and England are widely different in regard to the manner in which these enquiries are conducted. "In every case of a body found dead, whether known or unknown, whether of high or of low condition, a post-mortem examination is required by the court, as the foundation of all legal proceedings." Again: "There is not a single medico-legal case relating to the dead body, in which a post-mortem examination will not be of the highest service." (p. 339.) We have in these remarks the explanation why medical jurisprudence thrives more abroad than it does in England. It would, we think, be impossible to point out any other country in which we have the spectacle of some men devoting their lives to the cultivation of a science, the principles of which they are not permitted to practise; while others, who have confessedly never attended to the subject, are taken at random to answer questions involving the life or liberty of an accused party. The rule followed by our coroners in the very few cases in which they now require a medical opinion on the cause of death, is to summon that individual of the profession who happens to live *near* to the spot where the dead body has been found. Whether the witness so summoned be a surgeon or a druggist—a qualified or an unqualified practitioner—a master or an apprentice—whether he have had an opportunity of seeing such cases or not, and, therefore, whether he be competent or not to express an opinion as to the cause of death in this particular instance, are matters into which the coroner does not deem it necessary to enquire. Our professional brethren who have had any experience of coroners' inquisitions will, we think, bear us out in the statement that this is no exaggerated picture. Nor is it any answer to this accusation that coroners do sometimes select well-educated and qualified practitioners as witnesses. Our complaint is against the general rule that is followed, not against these occasional exceptions.

It is said that a knowledge of the cause will suggest a remedy for an evil. The cause in this case is easily discovered: it consists, we firmly believe, in the very defective manner in which individuals are appointed to the office of coroner. Our continental brethren will, perhaps, be astonished to learn that individuals are elected to this office in a way somewhat similar to that in which members of parliament are chosen to represent counties.* No qualification beyond that which pecuniary or other such influence can command, is required. The candidate need not possess the least knowledge of medicine or medical jurisprudence; it is not even necessary that he should have ever seen a dead body. We do not by these remarks wish to imply that a complete medical education is essential to one filling this office, although the proceedings in the large majority of inquests show that a much greater knowledge of medicine than of law, is really required on the part of this officer. General belief, however, appears to be so far in favour of the reverse, that most coroners are men who have been educated exclusively to the law. It is a favorite position with many persons that a sound knowledge of law is indispensable to the exercise of the office; but that this is a mistake is clearly proved by the fact that there is scarcely an inquisition in the proceedings of which, if it were necessary, a clever barrister could not easily detect many technical defects, that would lead to their being quashed before a superior tribunal. The credit for legal accuracy, therefore, which the law-proceedings of coroners' courts obtain in the world is really due to there being so rarely a necessity for disturbing them. Again, it is plausibly stated that the coroner can always command the best medical evidence. Not to mention that this position assumes a man to be well fitted to receive and judge of the value of evidence (in relation to the cause of death) on subjects of which he is totally ignorant, it is completely set aside by the facts: 1, that the *legal* coroner now rarely summons a medical witness before him; 2, when he does depart from this rule, he takes no care to inform

* The very lowest and most ignorant class of freeholders, who are not entitled to vote for members of Parliament, can vote on the election of a coroner!

himself how far that witness is competent to give *evidence* of *opinion.* Let us establish these two points before proceeding further.

*a.* By the 6 and 7 William IV. c. 89, the power of summoning medical witnesses is given to a coroner or a majority of the jury, and the witnesses are directed to be remunerated for their attendance. This was a considerable improvement on the old plan, in which a compulsory summons to attend was issued, without any remuneration being granted for the duty performed. The statute, however, led to this effect—inquests were now conducted without medical evidence being required so frequently as formerly; the option of summoning a witness being left with the coroner and jury. In the year 1837, another act was passed (1 Victoria, c. 68,) in which the expenses of medical witnesses were directed to be paid, in the first instance, by the coroner, to be repaid to him quarterly, at the General Sessions. This has turned out to be a most unfortunate enactment, since it has rendered the receiving of medical evidence at inquests a temporary burden on the coroner. We cannot be surprised, therefore, that these functionaries should take every opportunity of dispensing with the attendance of medical witnesses and with a post-mortem examination. Is it necessary for us to say more than this? Is it not within the knowledge of our readers that a large number of inquests are annually held in this country in which medical evidence is not demanded? We have now before us an authentic document, in the form of a Quarter Sessions' Report, by a magistrate of one of our midland counties; this report was made in October, 1838. We learn from it that out of the total number of inquests for the quarter (127), medical witnesses were summoned in 21 cases only, and therefore about *once* in *six inquests.* One coroner, it appears, held 82 inquests, and dispensed with medical evidence in 79 of these! The report proceeds to say, that "due regard should be paid to the expense entailed upon the county, from requiring such evidence; and due caution against incurring that expense without adequate reason"!

But it may be said there are many cases in which medical evidence is not necessary. We cannot give our assent to this statement. The duty of a coroner is to examine into the *cause of death:* if this be so clear as not to require a medical opinion, we cannot see the necessity for holding an inquest at all. The correctness of our views would be at once tested in paying the coroner by a fixed salary, and not by a fee upon every inquest which he holds. On the present system medical evidence is often not called for where disinterested persons would allow it to be necessary. Some cases of a recent occurrence will show this: A girl died in a low lodging-house, under circumstances which led to a strong medical suspicion that she had been poisoned by opium. An inquest was held—no medical evidence was required, and a verdict of "Died by the visitation of God" was returned. A man was found dead on the bank of a river, with marks of bruises and other violence on his person. The inquest was disposed of without medical evidence or a post-mortem examination, the coroner stating to the jury that it was most probable that the deceased was drunk; that the injuries might be ascribed to a fall; and that he was probably accidentally drowned. About a year afterwards some persons were arrested on a charge of having murdered the deceased; but when the case was heard before a magistrate, no material evidence could be brought against them, since the body had not been examined at the coroner's inquest; and the *real* cause of death was wholly unexplained. We might easily adduce many more such instances, but we think the occurrence of one case sufficient to show that the discretion now left to the coroner may be used to the direct impediment of the course of justice.

*b.* That witnesses, when summoned, are not selected, but taken without reference to their professional knowledge or experience, is a matter too well known to need illustration by examples. In some instances, where several witnesses have appeared, a coroner has actually balanced the numbers and decided by the numerical majority. Thus if four witnesses gave one opinion, and two another, a coroner has been guided by the first, because there was a majority of two in its favour; not considering that the evidence of one experienced man ought to be of more weight than that of ten who have had no experience on the subject. This proceeds from that officer being totally unacquainted with legal medicine; from his not knowing, for example, the difference in the symptoms produced by arsenic, opium, or carbonic acid; from his being unable to distinguish between a false and a true opinion, or between an inexperienced and an experienced witness. Let the case go before a higher tribunal for trial: the first question asked by the prisoner's counsel is, whether the medical witness has had any experience in similar cases; if not, which is most probable, an appeal is made to the jury, and sometimes with success, in the prisoner's favour. Now, we would ask, why, if such a question as this is allowed to be put in a superior court, should the summoning of witnesses before a coroner be a matter of pure accident? The witnesses at the inquest are commonly the witnesses at the trial; and if experienced men be required in the latter case, which the questions of prisoner's counsel seem to imply, why should they not be sought for by the coroner? This discretion left in the hands of the coroner becomes then a means of ultimately favouring the escape of the guilty.

Again: inquests are held unnecessarily, and the peace of private families is often intruded on in a most officious manner, more especially in cases of sudden death. Inquests are held in cases of apoplexy, where a little medical knowledge would inform the coroner that there could not be the remotest suspicion of poisoning: and thus we have constantly before us the empty verdict of "Died by the visitation of God," which leaves the enquiry exactly where it began. In other cases, from his want of knowledge, a coroner will set at defiance well-grounded medical opinions, and direct a verdict according to his own view of the medical circumstances. During the time of the cholera a coroner committed two persons for manslaughter, whereas all the medical witnesses, to the number of seven, deposed that the deceased had died of cholera. Before a superior tribunal the case was at once dismissed; but in the mean time the accused had been unjustly imprisoned, because the coroner could not distinguish between death from natural and violent causes, even where there were good opinions to guide him.

As inquests are at present conducted, a great source of knowledge is cut off from practitioners; there is, probably, not one in five hundred of the profession who has seen a drowned subject inspected. The reason is obvious: out of many hundred such cases occurring annually in or about the metropolis, the enquiry is generally conducted without medical evidence, and not one drowned subject in a hundred is submitted to a post-mortem examination by the order of a coroner. Then, again, suicidal cases of poisoning by oxalic or hydrocyanic acid are very frequent; but the body is rarely, if ever, permitted to be examined. The inquest is held, and either no medical opinion is sought for, or no inspection is made. If a trial for murder by drowning, or poisoning by oxalic or prussic acid come before a superior court, the medical evidence is either slighted or rejected, because the witness has not seen *that* which the coroner does not permit him to see. We consider the onus to rest with the coroner; for it is in the power of this officer alone to cause every body to be inspected.

Our readers will now be prepared for our conclusion, which is, that medical jurisprudence is extensively *taught* in England, and but little *practised.* The Apothecaries' Company have done much by enforcing attention to this subject; and the College of Surgeons have tardily recognized the necessity for its forming a part of the education of an English surgeon. But, notwithstanding the regulations issued, there is an indifference to the study—a circumstance which cannot surprise us, when we consider: 1. That teaching is not accompanied by practical expositions: medicine, surgery, chemistry, botany, all have the means of illustration; medical jurisprudence has not. 2. Students, in taking out a diploma, are not examined on the subject. 3. Future medico-legal practice is purely a matter of accident, no preference being given to one who has really studied the science.

The second defect we are glad to see remedied by the regulations recently adopted by the University of London. Strict examinations are instituted, and a nominal attendance at lectures is not held sufficient. At the same time, we wish it were in the power of the University to obtain for teachers the means of illustrating their lectures practically. This is done in France, Germany, and Italy; and, as it is admitted by all to be the only true way of teaching a science, we do not see why our own country should remain an exception to the general example of Europe.

The means by which, in our opinion, this science may be raised to the rank it deserves to hold are as follows: 1. That coroners should be educated men, acquainted with the principles of law and medicine; it matters not from which profession they be taken, but, to prevent any imputation of interested motives, we think they should not practise either. It may be said this is insisting upon too much; the judges of our superior courts are not versed in medicine, and yet they are called upon to try cases, involving medical facts and opinions. Our answer to this is, if coroners possessed only one half of the medico-legal knowledge possessed by judges, these objections to their competency would not exist. We wish to see the words of the ancient writ "*de coronatore eligendo*" rigorously adhered to: "*quod talem eligi faciat qui melius et sciat, et velit, et possit, illi officio intendere.*" If freeholders of every grade are to have the election of these officers, let their choice be at least restricted to persons who are fully qualified for the office; and it appears to us that in the present day there ought to be some other qualification than that of a man having "lands enough to be made a knight." 2. The salary paid to this functionary should not be regulated by the number of inquests which he holds. It is a premium upon their being held unnecessarily, a fact established by daily experience. We are glad to find ourselves supported by a high law authority in this view: Blackstone says (vol. i. 347), in relation to the coronership, "This office has been suffered to fall into disrepute, and get into low and indigent hands." Again: "For many years past they (coroners) have only desired to be chosen for the sake of their perquisites." (*Ib.*) 3. No inquest should be held without the evidence of a properly qualified and experienced medical practitioner being imperatively required; nor should a post-mortem examination be omitted. This rule ought to be adopted, if not as a matter of necessity, at least as one of sound policy, in order that good experience may be acquired by practitioners for occasions in which such experience is held to be essential to the due course of justice, namely, before the high tribunals of the land. This would not add to the expenses of the proceedings; for, so soon as the present improper mode of remunerating coroners was discontinued, fewer inquests would certainly be held; and those which were held, would be more carefully conducted.

We turn from this view of the state of medical jurisprudence in England to the work of our author; and we shall now proceed to examine his opinions on two other important subjects, Toxicology and Wounds.

10. The second volume is devoted exclusively to TOXICOLOGY, and the general history of poisoning, with the properties and distinguishing characters of each poison, are fully treated. Among the diseases of the stomach, which in their symptoms approximate to the general effects of irritant poison, perhaps scirrhous ulceration, leading to perforation, is the most striking and the most liable to mislead a practitioner. The following cases, given by the author, are interesting.

"A man, after a meal of dressed grain and new wine, was seized with a great desire to vomit; the abdomen became tense; there was the most violent pain in the stomach, and the skin was cold. There were great anxiety, impossibility to keep the recumbent posture, and difficulty of swallowing. The man died; and, on inspection, an aperture of about an inch in diameter was found near the lower orifice of the stomach, through which the contents of the organ had escaped into the abdomen. The margin of the aperture presented traces of scirrhous disease. The man had suffered from dyspepsia for some time previously. In another case a man, addicted to the use of spirituous liquors, was seized with the most severe pain in the abdomen and vomiting. A tumour appeared in the epigastric region, which evidently pulsated, and led the author to suspect that it was an aneurism of the abdominal aorta. The pain in the abdomen increased, but the tumour disappeared. The patient died; and, on inspection, there was no trace of aneurism, but the stomach was scirrhous and perforated." (Vol. ii. p. 52.)

These cases, which simulated poisoning, had their true nature explained by the long previous illness and by the post-mortem evidence, derived from the diseased state of the stomach. We agree that there are satisfactory means of diagnosis; but the author has omitted to state that a person suffering under such a disease of this organ may have poison administered to him, and die from its effects. A case of this kind occurred in the west of England lately, and created some difficulty in the medico-legal investigation. The discovery of arsenic in the stomach, with the symptoms produced by that poison, superadded to those under which the deceased had been already for some time suffering, served as the basis for a satisfactory diagnosis; and the prisoner was convicted on this evidence. The symptoms of Asiatic cholera may be mistaken for those of irritant poisoning. Our author thinks that a good diagnosis may be obtained by an examination of the blood; but we should rather trust to the character of the evacuations, and coldness and blueness of the surface. The general characters of irritant poisoning are well laid down. We must, however, distinguish between the action of irritant and corrosive poisons; for all irritant poisons are not corrosive. Corrosive poisons are known by their symptoms being produced *immediately;* they chemically destroy the organic tissues on contact; they give rise to the most violent pain and vomiting, while the evidence of their action after death is plainly discoverable, from the mouth to the stomach, if the person have not long survived the effects. These poisons not merely inflame, but corrode and perforate; whereas simple irritants are not often attended with either condition. From the description of symptoms by another, a practitioner may at once know whether the poison was a corrosive or not. "One remarkable circumstance must not be forgotten: these corrosive poisons do not always act on or destroy the parts with which they come in contact." (p. 62.) This last circumstance, though of rare occurrence, well deserves to fix our attention. Sulphuric acid has been sometimes swallowed without corroding the stomach; a fact referred by some to the acid being in small quantity, and becoming enveloped in mucus, which seemed effectually to prevent its contact with the parietes of the organ. The rules for preserving the identity of suspected liquids, as well as for conducting the analysis in unknown cases, are carefully described; and the necessity for previously determining the purity of the tests employed, rigorously insisted on. Much trouble will be spared by enquiring into the manner in which the deceased died, and into the nature of the symptoms under which he suffered previous to death. Thus we should not commonly look for arsenic in a case in which there had been no symptoms of irritation; nor for opium where there had been violent vomiting and diarrhœa, and no coma. When the poison has not been swallowed, of course these observations cannot apply.

The irritant poisons are examined in succession, beginning with the most important, as mercury, arsenic, &c. Each poison is systematically treated, so that any particular point connected with it may be at once found by a practitioner. In this respect Barzellotti's work is superior to that of any continental writer which we have yet seen. The history of the poison; its various forms; the symptoms and post-mortem appearances produced by it; the remedies or antidotes to be exhibited; a series of cases in which it has proved fatal; the chemical processes for detecting it; and, lastly, a general summary (epilogo dei fenomeni) of the pathological and chemical characters of the poison, render the toxicological history of each substance as complete as can be desired. One circumstance has surprised us, and created in our minds a feeling of disappointment. Out of the large number of cases reported in the volume, with but one or two unimportant exceptions, none are derived from the author's own observation, or from that of his countrymen. If we except a few ancient cases from Morgagni, nearly all are translated from Orfila. We have not met with above two or three from any modern Italian records. This appears to us singular, since instances of poisoning must, we should imagine, be as frequent in Italy as in France or England. We cannot, therefore, present any cases in illustration of the author's views: those of Orfila are so well known to English readers as to render it wholly unnecessary to quote them.

In poisoning by mercury, gluten is recommended as superior to albumen for its antidotal powers. (p. 100.) Taddei had for this reason proposed that it should be kept ready-prepared by apothecaries; but this is a matter of difficulty, and it appears to us that albumen is to be preferred, if for no other reason, at least for its being generally at hand, and requiring no time or complicated process, like gluten, to prepare and apply it. In administering antidotes the old motto of "*bis qui cito*" should be constantly borne in mind. There are various opinions as to the manner in which albumen operates in counteracting the effects of corrosive sublimate, and also with respect to the nature of the chemical compound formed. We certainly do not think the antidotal powers of albumen or gluten are to be explained by the fact of their forming insoluble compounds with this salt, because some substances, far less soluble in water than it, are capable of acting as energetic poisons, as, for instance, the arsenite of copper and the carbonates of lead, copper, and barytes.

The chemical analysis of this poison is very well described; but we think the tasting of corrosive sublimate and receiving its vapours into the nostrils and fauces may be dispensed with, since, while this is a dangerous method of experimenting, it does not add to the certainty derived from chemical tests. Differences of opinion will exist among medical jurists, as to the relative value of the processes for detecting poisons; but we shall say at once of this work that we think it, in the chemical department, superior in accuracy to those of Orfila and Devergie, although it is very closely modelled on the treatise of the former. After the very modest appeal to his readers, made by the author at page 682, we should not feel ourselves justified in criticising his remarks, even had we found much room for criticism, which we certainly have not. If, then, we make a few observations on some of his processes, we trust he will consider them more in the light of suggestions for improvement than of any attempt to depreciate what he has already done.

In the reduction of corrosive sublimate, carbonate of soda appears to us preferable to caustic potash, because it is more universally found, is more readily miscible, and is not deliquescent. As an antidote to arsenic, we find a solution of sulphuretted hydrogen recommended; but this seems to us an objectionable practice, since, if given in small quantity, it cannot decompose the poison in the manner alleged, and if given in large doses, it may itself act deleteriously. The hydrated peroxide of iron is also recommended, followed by an emetic; but Taddei has proved that the arsenite of iron, stated to be formed, is itself a poison. The opinions of English practitioners are much divided on the alleged antidotal properties of the hydrated peroxide. For ourselves, we have never seen any good effects from its use, and the most rational plan of treatment appears to us to be the application of the stomach-pump, or the timely use of an emetic, should not the poison itself operate as such. In describing the processes for detecting arsenic, the author represents this poison (arsenious acid) to have "an acrid, styptic taste, and to excite salivation." (p. 162.) This appears to us to be a mistake; it is well known that many persons who have been poisoned by it have not observed any particular taste. Besides, Dr. Christison has shown that this supposed acrid taste is founded on popular error. The use of chlorine or animal charcoal to decolorize organic liquids suspected to contain this poison, is very properly denounced by the author. The colouring matter presents no obstacle to the safe and certain action of sulphuretted hydrogen gas. The copper test should be rejected in all coloured liquids. In the event of our not finding arsenic in the viscera, we are recommended to examine the blood, lymph, and other secretions. We believe arsenic has not yet been detected in the blood of persons poisoned by it; we have frequently sought for it by the delicate "hydrogen test," but without success.

There is but little to remark in relation to the other metallic irritants. Albumen is recommended as an antidote in poisoning by copper; but it is not stated that the albuminate of copper formed, is readily soluble in an excess of the poisonous salt: a fact of some importance in the treatment, since it shows that for this substance to act with any prospect of benefit it must be exhibited in very considerable quantity. A strong characteristic of poisoning by copper is that the salts give a deep blue or green colour to the substances with which they are mixed, as well as to the vomited matters. In analysing the contents of a stomach, we must remember that sulphate of copper is sometimes given as an emetic, and very largely; a circumstance which might complicate the analysis, were we not prepared for it. The professor dwells much, chiefly on the authority of others, on the presence of traces of copper in most organic substances used as food. We have already, we think, disposed of this apparent difficulty in one of our late Numbers, in some remarks on the blood. To discover antimony we are recommended to calcine the precipitates obtained by the tests "with a mixture of potash and charcoal, in a *platina* crucible." (p. 219.) Surely this must be an oversight: antimony, in a metallic state, combines and forms so fusible an alloy with platina, that the crucible would be destroyed, and the antimony lost, if any were present. Oxalic acid probably is not so much used as a poison in Italy as in England; at least this is the only way in which we can account for the few lines devoted to its history and properties. The process for detecting this, to us, important poison, is not so satisfactorily given as we could wish to see. Considerable space is occupied by an account of the vegetable irritants, and of the best chemical means which we have at hand to discover the presence of the active alkaline principles to which they owe their poisonous effects.

The narcotic and narcotico-irritant poisons are next disposed of. The author here, as in the other parts of his volume, seems to have consulted every available source. In treating of opium, he objects to Christison's view of directing our analytical researches to the discovery of meconate of morphia alone; but we do not think his objection valid or of any practical force. It is not likely that meconate of morphia would be found in the human stomach without some strong suspicion or even circumstantial evidence of opium having been taken by the deceased. The discovery of the meconate would then be all that the law requires or ought to require. If we are to take the author's view, we ought to obtain narcotine, narceine, and codeine; but we might as reasonably go a step further, and insist upon the resin, gum, tannin, and colouring matter, with other vegetable principles, being reproduced, before speaking to the presence of the drug. Such a refinement is not, in the present day, possible of application; and even if it were we should say that it would be unnecessary. The history of poisoning by hydrocyanic acid is very complete; but we certainly should not recommend the tasting of the poison as one step in the analysis. The author frequently subjoins to his description of the individual poison the mode of determining its quantity, a necessary kind of information to the medical jurist, which is not commonly met with in toxicological works. To the history of each class of poisons is appended a useful Table, containing a brief summary for each substance of the information contained in the foregoing chapters. Let us notice another excellent peculiarity of this volume, which is, that the subject of compound poisoning, one of considerable difficulty to the medical jurist, is discussed with brevity and clearness. The author acknowledges that he has taken the idea of this from Orfila.

The second volume is concluded with an examination of a very important question, viz.: how far those engaged in the general practice of the profession should be permitted to conduct investigations in toxicology? In examining this question the author repudiates the idea of throwing the least censure upon the general acquirements of his professional brethren; but he contends that these are, by the system of education adopted, chiefly directed to one object alone—namely, that of fitting a man to practise medicine and surgery. "Unless the attention of a student be earnestly and assiduously given to the study of chemistry, he is as likely to be unacquainted with the niceties of this science as with those of mathematics or astronomy." (p. 672.) "A man may be clever in his profession, but how is he to have at command the knowledge of a subject which he has never studied?" If this be true, courts of law ought not to receive their evidence in the same way as they would receive that of others who were well skilled in the science. To support these views he selects some examples in illustration of what a medical witness may have to do. "The analysis of corrosive sublimate is a very simple matter when it is presented in a pure and unmixed state; but when it is not found either internally or externally, how is the suspicion of poisoning to be supported or rebutted by the practitioner? Mercury may, however, be found in the body in the state of calomel, or oxide, or pure metal; and how is an opinion to be expressed here as to the cause of the symptoms, or the probability of the poison having been decomposed by the organic tissues? Either by showing the corrosive action on the viscera or by discovering the poison in a state of combination with organic matter, a process far from easy." (p. 675.) "But supposing that none of the poison is found, and there are no marks of its action on the living body, are we to deny the fact of poisoning?" Before an answer can be returned to this question, many minute circumstances must be taken into consideration, which will require a large share of experience on the part of the practitioner. In compound poisoning, how will the analysis be conducted, except by one who has at his command all the resources of science? Opium furnishes another example of difficulty to a medical witness who attempts to search for it without being well versed in chemistry. But here the author thinks that it is not enough to detect meconate of morphia; he must be able to separate the other principles, and identify them by their properties, before he proceeds to infer that opium was present in the substance analysed. If this were indispensably necessary, we do not think there is one chemist in a thousand who would be competent to the task. Nux vomica presents another case of complex analysis. "This poison contains two principles, strychnia and brucia, which have not hitherto been found united in any other substance." (p. 679.) To justify an opinion that nux vomica was present it is not sufficient to show strychnia only, or brucia only; for the one exists in the bean of St. Ignatius, and the other in the false Augustura. Both of these principles must be produced—they must be separated from each other, and their properties respectively demonstrated. We agree with the author that but few of those engaged in general practice would be competent to perform such a duty; but at the same time we think there are few professed chemists who would succeed, unless they had specially directed their attention to the separation of the alkaloids from vegetables, and had acquired considerable experience in this department. The fourth illustration is an unfortunate one for the position assumed, since it goes against the wants of science generally, not against those who profess it. When a person has died from the effects of sulphuretted hydrogen gas, it is said the cause of death cannot be determined, since there is no poison capable of detection left within the body. The odour of the gas in the body would not furnish sufficient evidence, because in putrefaction the same odour is given out; but we think a case of this kind is easily disposed of. Death from sulphuretted hydrogen is the result of accident or homicide; in either case the body is found in the infected spot, and circumstantial evidence supplies that which was defective in a medical view. Besides, it by no means follows that the bodies of all who are destroyed by sulphuretted hydrogen should be found in a state of putrefaction.

Although we admit that there is a great deal of truth in the author's remarks on this subject, we cannot agree with him that these investigations should be taken out of the hands of general practitioners. It would entirely do away with the teaching of medical jurisprudence; for who would learn a science, the practice of which would be confined to a few? Our opinion is that the practical study of the subject should be enforced on all, and that the means of acquiring a sound knowledge of it should be freely at the disposal of every one who enters the profession. Nothing, as we have endeavoured to show, can be worse than the present system in England, in which individuals are summoned to give evidence, not simply of facts, but of *opinion*, whether they have been three months or thirty years in the profession; and evidence of the composition of a suspected poisonous liquid is taken from one who perhaps never attempted an analysis before. In spite of the discouragement thus given to those practitioners who have attended to this department of science, it is certain that a very great improvement has taken place of late years in medical evidence relating to poisoning. There are few of the modern school who are not competent to detect arsenic and some of the more common poisons readily, even under circumstances of difficulty; and we may hope that a few years will enable others to advance still further in the practice of toxicology. If the plan recommended by the author were adopted there would be an end to all further improvement in medico-legal knowledge in the great body of the profession; but we frankly admit with him that, as the case at present stands, our legal authorities are bound to give a preference to those who have studied the subject over those who have not.

11. The first half of the third volume is devoted to the subject of WOUNDS and other local injuries, which the author treats differently to most other medico-legal writers. He gives to this branch the name of Surgical Jurisprudence (Chirurgia Forense). A large space is occupied by the account of injuries to different parts of the body; and a peculiarity of his plan is that he subjoins the surgical treatment of these injuries, which, in our opinion, is passing rather beyond the true bounds of the science. A large number of cases are added, chiefly derived from Morgagni, Uccelli, and Boyer. These cases are not so much adapted to illustrate medico-legal doctrines and opinions as the rationale and results of surgical treatment. They are mostly hospital cases, not cases which have come before the courts for investigation or trial.

The author defines a wound to be "a breach of continuity in the organic tissues violently produced through external causes." (p. 16.) This definition would of course bring fractures and dislocations within the meaning of wounds, a position which ought to be adopted in legal medicine, although most surgeons would dissent from it, as applied to surgery. In speaking of *ecchymosis*, he complains of Orfila for rejecting the word *sugillation*, as it is used to designate certain forms of ecchymosis, depending on internal causes. (p. 31.) We cannot conceive, however, on what consistent grounds this word "sugillation" is to be retained. The term ecchymosis expresses all that is required in legal medicine.

We do not intend to follow our author in his account of injuries to the different parts of the body. The following case shows what serious mischief may proceed from a slight local injury: "An old man, who was stealing wood, was suddenly surprised by the proprietor, when, after some words between them, the latter struck him on the back with a stick; the old man ran two or three paces, and then fell dead. On examination there was no mark of external violence, but internally the aorta was found ruptured. This was the cause of death." (p. 109.) One of the most common consequences of a wound of the bladder is immediate extravasation of urine, from which death takes place in about a week. "A medical student was shot through the bladder and rectum in a duel. He did not die until the twentieth day, and for the first few days no extravasation took place. This happens in gun-shot wounds only, when the sloughs produced by the wound fall off." (p. 174.)

A wounded man may die either within a short time or at a long period after having received the injury. In either case death may not depend on the wound, for the person may have been at the time labouring under disease, or circumstances may have sprung up to aggravate the effects of a comparatively slight injury. Serious questions of responsibility on the part of the aggressor will arise in such a case, and much will depend on the medical evidence given. It is well known that many subtle distinctions are made in the laws of continental countries, regarding the relative mortality of wounds. The following case shows, in our opinion, the ill effects of such distinctions : it is quoted by Barzellotti.

"A woman, one evening, received a violent blow on the head, which felled her to the ground, in a state of insensibility. She remained in this condition all night, senseless, and without assistance, and the next day she died. On inspection, the skull was found fractured, and there was a large extravasation of coagulated blood under the dura mater. The medical witnesses pronounced the wound to have been absolutely mortal; but the court, disregarding their opinions, and, guided by the dictates of reason and common sense, acquitted the aggressor of homicide, simply because the deceased was not assisted, as she ought to have been, by professional men. Trephining might here, as in other cases of wounds of the head, accompanied by extravasation, have saved life. It is of no avail to say that the deceased was struck while out of the town in the evening, and that the gates were shut. Circumstances of this kind, although to the detriment of the patient, must not be charged to the responsibility of the aggressor. If a practitioner had been procured, then only, when the best means of art had failed in recovering the deceased, should we have been in a condition to judge of the mortality of the wound." (Vol. iii. p. 263.)

We are surprised how a doctrine of this kind can be set down as consistent with reason and common sense. Here is an injury inflicted, always serious, and commonly fatal, even under the best treatment timely rendered. The deceased did not seek for medical assistance, simply because it was impossible for her to do this, the murderous violence having deprived her of the power, by rendering her insensible. It was not to be expected that the murderer himself should fetch a professional man, and it cannot be supposed that medical practitioners are to be stationed about high roads, with their instruments, looking out for cases of this kind! Medical assistance could not then possibly be afforded to the deceased ; but how can it affect the moral or legal responsibility of an aggressor in such a case as this, whether medical assistance be at hand or not ? Our opinion is that the presence of a medical man with his instruments, when the wound is inflicted, does not diminish the crime, or lessen, in the smallest degree, the responsibility of the accused. Wounds of the carotid artery have not always proved fatal, owing to the timely application of a ligature. But if a man divide the carotid artery of another, and the wounded person die, is the aggressor to be acquitted of homicide because, had a surgeon been at hand, he might have tied the artery and saved life ? Reason will show that, if such a position be once admitted, the responsibility of those who inflict murderous wounds may be, in most cases, easily frittered away by abstract medical speculations.

The characters of *vital* and *post-mortem* wounds are elaborately described. Some space is devoted to the subject of spontaneous combustion, a doctrine which our author seems inclined to favour (p. 285); but as he would not give an opinion in the affirmative until, among other proofs, it were shown that the burning could not have taken place from communication with ignited matter, we willingly join him in the degree of credit which he virtually attaches to this alleged phenomenon. The remarks on the chemical examination of blood-stains are practical and valuable. Some interesting cases are related, in which the importance of these enquiries are well set forth. The responsibility of aggressors, as it is affected by necessary surgical operations on the wounded—the numerous circumstances which aggravate the effects of local injuries, and the means of identifying wounds by their cicatrices, are subjects ably treated in the remaining chapters.

We here close our notice of this work, having confined it to the strictly medico-legal portion. The last half of the third volume treats of contagion, and medical police regarding contagious diseases. We may reserve our remarks on this until another opportunity, when the subject of medical police comes before us. We have, in a great degree, anticipated our judgment of the whole treatise. If we regard matter, system in the individual parts, and comprehensiveness in the whole, we think it, without exception, the best on medical jurisprudence that has issued from the continental press. The subjects are treated with as much judgment and ability as the mind of any man can be supposed capable of bringing to a science which demands not only an extensive knowledge of every branch of medicine, but of the principles of jurisprudence. One rare merit this work possesses over those of many French and German authors—in no part have we met with anything like dogmatism. The writer states his opinions firmly and modestly; and this is no slight praise to him, considering that of all sciences there is none so apt to lead its followers to dogmatize, because they may, in many instances, dogmatize with impunity. An error propagated on great authority may, it is well known, remain many years undetected; and, in the meantime, lead to irreparable mischief. We have not hesitated to state freely our opinion of some of the doctrines of Professor Barzellotti, but we think this will be no ground of displeasure to one whose object has been evidently throughout to write for the sake of truth, and not for victory. We have endeavoured to do justice, at the same time, to our author and our readers; to show that what we considered faults did not escape our notice. But in executing this task we trust we have always borne in mind the noble observation quoted by him from his countryman Redi, "*Siamo uomini e per conseguenza soggetti ad errare.*"

---

